# Chinese pediatrician beliefs about counseling and medications for parents who smoke: a survey in southern China

**DOI:** 10.1186/s12971-015-0035-x

**Published:** 2015-04-07

**Authors:** Kaiyong Huang, Abu S Abdullah, Jing Liao, Haiying Huo, Li Yang, Zhiyong Zhang, Jonathan P Winickoff, Guangmin Nong

**Affiliations:** School of Public Health, Guangxi Medical University, Nanning, Guangxi 530021 China; Global Health Program, Duke Kunshan University, Duke Avenue, Kunshan, Jiangsu 215316 China; Duke Global Health Institute, Duke University, Durham, North Carolina USA; Boston University School of Medicine, Boston Medical Center, Boston, Massachusetts USA; Department of Pediatrics, The First Affiliated Hospital of Guangxi Medical University, Nanning, Guangxi 530021 China; MGH Center for Child and Adolescent Health Research and Policy, Harvard Medical School, Boston, USA

**Keywords:** Pediatrician, Beliefs, Effectiveness, Smoking cessation, Counseling, Medication

## Abstract

**Background:**

Pediatricians play an important role in promoting smoking cessation among the parents of young children as more behavioral counseling and cessation treatment are made available in the Chinese healthcare system. However, beliefs about the effectiveness of these interventions can influence pediatricians’ recommendations to their patients. This study examined pediatricians’ beliefs regarding effectiveness of counseling and medications for smoking cessation.

**Methods:**

A cross-sectional survey of pediatricians was conducted in thirteen conveniently selected southern Chinese hospitals, during September to December 2013. A self-administered questionnaire was used for data collection. We used chi square tests and multinomial logistic regression analysis to identify factors associated with beliefs regarding effectiveness of counseling and medications for smoking cessation.

**Results:**

Beliefs of the respondents (504/550; 92% response rate) were divided regarding the effectiveness of counseling and medications for smoking cessation. Sixty percent believed that physician counseling is effective for smoking cessation; 53% believed pharmacological products (or medications) are effective. Factors that were associated with positive beliefs towards the effectiveness of counseling included: believing about the professional responsibility to discuss smoking cessation, being confident in discussing smoking cessation or SHS exposure reduction with patients’ parents, believing that health professionals should routinely ask about their patients smoking habits, believing that health professionals should routinely advise their smoking patients to quit smoking, possessing adequate knowledge in quitting smoking, and being able to assess smokers different stages of readiness to quit. Most of the above factors were also associated with the belief that medication is effective for smoking cessation.

**Conclusions:**

A substantial proportion of Chinese pediatricians believed that cessation counseling and medications are not effective, which is not supported by current evidence in the field. Several factors including individual, practice level and health system level characteristics were associated with their belief. Training efforts are needed to influence pediatricians’ beliefs regarding the effectiveness of cessation counseling and medications.

## Background

China is the world’s largest producer and consumer of tobacco with 350 million smokers [1]. China has 740 million non-smokers passively exposed to SHS, including 180 million children under the age of 15 [2,3]. At present, an estimated 1 million deaths from smoking occur in China each year, and if the current smoking rates continue, as many as 100 million people currently under the age of 30 in China will die from tobacco use [1,2]. Thus, it is clear that tobacco control efforts in China must be intensified to combat the tobacco-induced morbidity and mortality.

Smoking cessation is a priority for preventing smoking-attributable disease and reducing its burden [4,5]. However, promotion of smoking cessation counseling and cessation medications by physicians is an obstacle for China [6]. Among the physicians group, pediatricians who can address both secondhand smoke (SHS) exposure to children as well as parental smoking cessation are in a critical position. Because, the SHS exposure to children due to parental tobacco use is a serious and prevalent health issue, with over twenty-four percent of children living in a home with a smoking parent [7]. Parental smokers often see their child’s doctor more frequently than their own, with an average of over 10 visits in the first two years of a child’s life [8,9]. However, very small proportions of pediatrician’s visits by a smoker result in cessation counseling or prescription for an effective smoking cessation medication [10]. It was argued that physicians are more likely to recommend treatments that they believe are effective [11]. In studies among physicians in overseas, belief regarding the effectiveness of counseling and cessation medications varied [12-14] with a substantial proportion holding misconceptions about the intervention effectiveness [13,14]. An exploratory study among Chinese pediatricians also reported misconceptions about the effectiveness of counseling and medications for smoking cessation [15]. Therefore, it is critical that the Chinese pediatrician’s beliefs are appropriate and evidence-based as they play a key role in clinical decision making. This study describes the beliefs of a sample of Chinese pediatricians regarding the effectiveness of counseling and medication for tobacco dependency treatment.

## Methods

### Sample

Participants were pediatricians working in the conveniently selected thirteen hospitals (twelve grade III and one grade I) in four major cities of Guangxi province (a Southern Chinese province), the People’s Republic of China.

### Data collection

A standardized Mandarin Chinese language questionnaire was used for data collection. Questionnaires were distributed through the director of pediatrics department in each of the participating hospitals. The director distributed a copy of the questionnaire to each pediatrician working in his or her department and requested them to put the completed questionnaire in a sealed envelope and drop the questionnaire in the designated box kept in the doctor’s office. Our study coordinator then collected the sealed questionnaire from each of the directors. For clarity on any unfinished questions, our study coordinator contacted the individual pediatrician by telephone. To compensate for their time, each participant was given a cash amount of RMB 100 ($15). The study was approved by the institutional review board of Guangxi Medical University.

### Questionnaire

The questionnaire was developed with reference to the questionnaires previously used by the investigators team in the United States [9] and in China [6]. The questionnaire obtained demographic information on the subject’s demographic background (sex, age, physician type, number of years studied at medical school), smoking behavior (smoker, non-smoker) and other questions on “counseling practices for smoking cessation and secondhand smoke (SHS) exposure to children”, “perceiving barriers for smoking cessation service”, “whether their workplace is smoke-free”, “whether they received any training on smoking cessation counseling”, which should be answered “yes” or “no”.

Belief of effectiveness for smoking cessation counseling was assessed by asking “physician counseling about smoking cessation is a cost-effective intervention compared to other preventive interventions?”, with response categories of “strongly agree, agree, disagree and strongly disagree”. Belief of effectiveness for pharmacological products was assessed by asking, “pharmacological products or medications (i.e., nicotine patch, nicotine gum, nicotine lozenges, bupropion, varenicline) are effective in helping smokers quit smoking”, with response categories of “strongly agree, agree, disagree and strongly disagree”. And responses were categorized as 1 for “strongly agree and agree”, 2 for “disagree and strongly disagree”.

### Analyses

Two members of the research team coded each questionnaire and entered all data with Epidata 3.1, and then made a data consistency check. We used *χ*^*2*^ procedures to compare differences between the belief of effectiveness and demographic characteristics or other relevant variables, and then used multinomial logistic regression analysis to analyze the factors with p-value <0.2 in *χ*^*2*^ procedures. A p-value of <0.05 (two-tailed) was considered statistically significant.

## Results

### Demographic and other characteristics

A total of 550 questionnaires were handed out and 504 pediatricians completed the questionnaires, with a response rate of 92% (504/550). Response rates were almost identical in all the hospitals. Of the respondents, 64% were female, 77% received 5 years of education at medical school and 17% were current smokers (Table 1). More than one-third of the respondents didn’t hear about e-cigarettes. As a non-smoker, 46% of all pediatricians inhaled secondhand smoke for more than 15 minutes daily for more than 1 day during the past week; 81% of the samples didn’t receive formal training in smoking cessation and 64% of all pediatricians didn’t read China smoking cessation guidelines. Of the respondents, 60% and 53% believed that physician counseling and pharmacological products are effective for smoking cessation, respectively.

**Table 1:** **Demographic and other characteristics of survey sample, Guangxi, China 2013 (n = 504)**

**Variables**	**N**	**%**
**Demographic and work environment characteristics**		
***Gender***		
Male	182	36
Female	322	64
***Age***		
20-30	215	43
31-40	159	31
41-50	89	18
Above 50	41	8
***Physician type***		
Resident Physician	223	45
Attending Physician	151	30
Associate Chief Physician	88	17
Chief Physician	42	8
***Number of years studied at medical school***		
5 Years	388	77
More than 5 years	116	23
**Tobacco use related characteristics**		
***Smoking status***		
Current smoker	82	17
Nonsmoker	400	83
***Use other forms of tobacco***		
No	480	95
Yes	24	5
***Heard about e-cigarettes***		
No	178	35
Yes	326	65
Exposed to SHS regularly*		
No	247	54
Yes	211	46
***Received cigarettes as gift or gave cigarettes as gifts to others***		
No	423	84
Yes	81	16
**Hospital policy characteristics**		
***Have smoke-free policy in the hospital***		
No policy	8	2
Have policy	496	98
***Hospital have any policy to advise smokers to quit***		
No	219	43
Yes	285	57
**Training and work attitudes**		
***Received formal training in smoking cessation***		
No	399	81
Yes	96	19
***Have read China smoking cessation guidelines***		
No	322	64
Yes	77	15
Never heard about it	105	21
***Have read international (i.e. US, UK) smoking cessation guidelines***		
No	359	71
Yes	36	7
Never heard about it	109	22
***Other international guidelines are useful***		
No	4	10
Yes	31	78
Not Sure	5	12
***Believe about the professional responsibility to discuss smoking cessation***		
Pediatricians	229	25
Nurses	160	18
The parent’s primary care doctor	379	41
Others	150	16
***Level of confidence discussing smoking cessation or SHS exposure reduction with patients’ parents***		
Not at all confident	66	13
Somewhat confident	316	63
Very confident	122	24
***Beliefs regarding effectiveness of physician counseling for smoking cessation***		
Agree/strongly agree	304	60
Disagree/strongly disagree	200	40
***Beliefs regarding effectiveness of pharmacological treatment for smoking cessation***		
Agree/strongly agree	266	53
Disagree/strongly disagree	238	47
***Lack of professional training in the area of tobacco cessation counseling is a major barrier***		
Yes	455	94
No	27	6

Note: Due to the missing values in some variables, the total number may not equal to the same. *exposed to SHS for more than 15 minutes daily for more than 1 day in the past week.

### The belief that physician counseling is effective for smoking cessation

Table 2 describes the pediatrician’s beliefs regarding effectiveness of smoking cessation counseling. A significantly higher proportion of female (64%) than male (54%) pediatricians believed that cessation counseling is effective (p < .05). A higher proportion of pediatricians who reported that their hospital have a policy to advise smokers to quit smoking (64%) than those which have no policy (55%) believed that cessation counseling were effective (p < .05). Pediatricians who received formal training in smoking cessation (71%) than those who did not receive training (58%) believed that cessation counseling were effective (p < .02). A significantly higher proportion of those who had read China smoking cessation guidelines (66%) than those who did not hear about the guidelines (49%) believed that cessation counseling is effective (p < .05). Other significant characteristics related to the belief that cessation counseling is effective included being confident (65%) than not at all confident (32%) in discussing smoking or SHS exposure reduction with parents, believing that pediatricians can help parents stop smoking (77%) than not believing so (40%), in the usual practice advising (64%) than not advising (17%) parents not to smoke around children, agreeing (65%) than not agreeing (37%) with the statements that health professionals should routinely ask about parents smoking habits, believing (69%) than not believing (27%) that health professionals should advise smoking parents to quit smoking, believing that they possess adequate knowledge (81%) than not believing so (56%) to help parents in quitting smoking, and those who could assess smokers different stages of readiness to quit (75%) than those who do not believe so (58%).

**Table 2:** **Factors associated with the belief that physician counseling is effective for smoking cessation, Guangxi, China 2013**

**Variables**	**Agree/strongly agree**	**Disagree/strongly disagree**	***χ*** ^***2***^	***P*** **value**
	**n (%)**	**n (%)**		
Total	304 (60)	200 (40)		
**Gender**				
Male	98 (54)	84 (46)	4.984	0.026
Female	206 (64)	116 (36)		
**Ages**				
20-30	126 (59)	89 (41)	1.977	0.577
31-40	93 (58)	66 (42)		
41-50	59 (66)	30 (34)		
Above 50	26 (63)	15 (37)		
**Physician type**				
Resident Physician	121 (54)	102 (46)	6.825	0.078
Attending Physician	95 (63)	56 (37)		
Associate Chief Physician	60 (68)	28 (32)		
Chief Physician	28 (67)	14 (33)		
**Number of years studied at medical school**				
1-5 Years	232 (60)	156 (40)	0.193	0.660
More than 5 years	72 (62)	44 (38)		
**Smoking status**				
Current smoker	51 (62)	31 (38)	0.108	0.743
Nonsmoker	241 (60)	159 (40)		
**Use other forms of tobacco**				
No	289 (60)	191 (40)	0.031	0.861
Yes	15 (63)	9 (37)		
**Heard about e-cigarettes**				
No	105 (59)	73 (41)	0.203	0.625
Yes	199 (61)	127 (39)		
**Exposed to SHS regularly**				
No	158 (64)	89 (36)	1.076	0.299
Yes	125 (59)	86 (41)		
**Received cigarettes as gift or gave cigarettes as gifts to others**				
No	251 (59)	172 (41)	1.055	0.304
Yes	53 (65)	28 (35)		
**Hospital have any policy to advise smokers to quit**				
No	121 (55)	98 (45)	4.153	0.042
Yes	183 (64)	102 (36)		
**Received formal training in smoking cessation**				
No	231 (58)	168 (42)	5.416	0.020
Yes	68 (71)	28 (29)		
**Have read China smoking cessation guidelines**				
No	201 (62)	121 (38)	6.833	0.033
Yes	51 (66)	26 (34)		
Never heard about it	52 (49)	53 (51)		
**Have read international (i.e. US, UK) smoking cessation guidelines**				
No	218 (61)	141 (39)	1.169	0.557
Yes	24 (67)	12 (33)		
Never heard about it	62 (57)	47 (43)		
**Other international Guidelines are useful**				
No	3 (75)	1 (25)	0.361	0.835
Yes	21 (68)	10 (32)		
Not sure	4 (80)	1 (20)		
**Believe about the professional responsibility to discuss smoking cessation**				
Pediatricians	159 (69)	70 (31)	34.927	0.000
Nurses	107 (67)	53 (33)		
The parent’s primary care doctor	250 (66)	129 (34)		
Others:	63 (42)	87 (58)		
**Level of confidence discussing smoking cessation or SHS exposure reduction with patients’ parents**				
Not at all confident	21 (32)	45 (68)	25.771	0.000
Very/Somewhat confident	283 (65)	155 (35)		
**Believe pediatricians can help patients’ parents to stop smoking**				
Agree/strongly agree	216 (77)	65 (23)	72.683	0.000
Disagree/strongly disagree	88 (40)	135 (60)		
**I am not familiar with the guidelines for stop smoking**				
Agree/strongly agree	181 (59)	128 (41)	1.012	0.314
Disagree/strongly disagree	123 (63)	72 (37)		
**Smoking cessation counseling for my patients’ parents is not an efficient use of my time**				
Agree/strongly agree	146 (57)	110 (43)	2.347	0.126
Disagree/strongly disagree	158 (64)	90 (36)		
**I am unaware of the best strategies for helping my patients’ parents to stop smoking**				
Agree/strongly agree	194 (62)	119 (38)	0.955	0.329
Disagree/strongly disagree	110 (58)	81 (42)		
**Advise patients who smoke to avoid smoking around children**				
Agree/strongly agree	298 (64)	171 (36)	29.291	0.000
Disagree/strongly disagree	6 (17)	29 (83)		
**Health professionals should routinely ask about their patients smoking habits**				
Agree/strongly agree	275 (65)	150 (35)	21.816	0.000
Disagree/strongly disagree	29 (37)	50 (63)		
**Heath professionals should routinely advise their smoking patients to quit smoking**				
Agree/strongly agree	275 (69)	123 (31)	60.920	0.000
Disagree/strongly disagree	29 (27)	77 (73)		
**My current knowledge is sufficient for helping patients to stop smoking**				
Agree/strongly agree	70 (81)	17 (19)	17.823	0.000
Disagree/strongly disagree	234 (56)	183 (44)		
**I can assess a smoker’s different stages of readiness to quit**				
Agree/strongly agree	61 (75)	21 (25)	8.103	0.004
Disagree/strongly disagree	243 (58)	179 (42)		

Multinomial logistic regression analysis showed that “believing about the professional responsibility to discuss smoking cessation”, “being confident in discussing smoking cessation or SHS exposure reduction with patients’ parents”, “believing that health professionals should routinely ask about their patients smoking habits”, “believing that health professionals should routinely advise their smoking patients to quit smoking”, “possessing adequate knowledge in quitting smoking”, and “being able to assess smokers different stages of readiness to quit” were the factors associated with the belief that physician counseling is effective for smoking cessation (see Table 3).

**Table 3:** **Multinomial logistic regression analysis on factors associated with the belief that physician counseling is effective for smoking cessation**

**Variables**	**Agree/strongly agree**	**Disagree/strongly disagree**	**Odds ratio**	***P*** **value**
	**n (%)**	**n (%)**	**(95% confidence interval)**	
**Believe about the professional responsibility to discuss smoking cessation**				
Pediatricians	159 (69)	70 (31)	1.855	0.042
Nurses	107 (67)	53 (33)	(1.024–3.362)	
The parent’s primary care doctor	250 (66)	129 (34)		
Others:	63 (42)	87 (58)		
**Level of confidence discussing smoking cessation or SHS exposure reduction with patients’ parents**				
Not at all confident	21 (32)	45 (68)	3.800	0.005
Very/Somewhat confident	283 (65)	155 (35)	(1.510–9.566)	
**Health professionals should routinely ask about their patients smoking habits**				
Agree/strongly agree	275 (65)	150 (35)	3.395	0.002
Disagree/strongly disagree	29 (37)	50 (63)	(1.591–7.244)	
**Heath professionals should routinely advise their smoking patients to quit smoking**				
Agree/strongly agree	275 (69)	123 (31)	4.129	0.000
Disagree/strongly disagree	29 (27)	77 (73)	(2.100–8.120)	
**My current knowledge is sufficient for helping patients to stop smoking**				
Agree/strongly agree	70 (81)	17 (19)	6.015	0.000
Disagree/strongly disagree	234 (56)	183 (44)	(2.366–15.293)	
**I can assess a smoker’s different stages of readiness to quit**				
Agree/strongly agree	61 (75)	21 (25)	3.613	0.003
Disagree/strongly disagree	243 (58)	179 (42)	(1.540-8.475)	

### The belief that pharmacological products are effective for smoking cessation

Table 4 describes that nine factors were associated with pediatrician’s beliefs regarding the effectiveness of pharmacological products for smoking cessation. These factors are: receiving (65%) than not receiving (50%) formal training in smoking cessation (p < .02), reading (66%) than not reading (53%) China smoking cessation guidelines (p < .01), being confident (55%) than not at all confident (38%) in discussing smoking or SHS exposure reduction with parents (p < .01), believing that pediatricians can help parents stop smoking (67%) than not believing so (35%) (p < .001), in the usual practice advising (55%) than not advising (23%) parents not to smoke around children (p < .001), agreeing (59%) than not agreeing (29%) with the statements that health professionals should routinely ask about parents smoking habits (p < .001), believing (61%) than not believing (24%) that health professionals should advise smoking parents to quit smoking (p < .001), believing that they possess adequate knowledge (74%) to help parents in quitting smoking than not believing so (48%) (p < .001), and those who could assess smokers different stages of readiness to quit (67%) than those who do not believe so (50%) (p < .01).

**Table 4:** **Factors associated with the belief that pharmacological products are effective for smoking cessation, Guangxi, China 2013**

**Variables**	**Agree/strongly agree**	**Disagree/strongly disagree**	***χ*** ^***2***^	***P*** **value**
	**n (%)**	**n (%)**		
Total	266 (53)	238 (47)		
**Gender**				
Male	86 (47)	96 (53)	3.489	0.062
Female	180 (56)	142 (44)		
**Ages**				
20-30	107 (50)	108 (50)	3.756	0.289
31-40	93 (59)	66 (41)		
41-50	43 (48)	46 (52)		
Above 50	23 (56)	18 (44)		
**Physician type**				
Resident Physician	116 (52)	107 (48)	3.392	0.335
Attending Physician	84 (56)	67 (44)		
Associate Chief Physician	49 (56)	39 (44)		
Chief Physician	17 (41)	25 (59)		
**Number of years studied at medical school**				
1-5 Years	207 (53)	181 (47)	0.222	0.638
More than 5 years	59 (51)	57 (49)		
**Smoking status**				
Current smoker	42 (51)	40 (49)	0.251	0.616
Nonsmoker	217 (54)	183 (46)		
**Use other forms of tobacco**				
No	251 (52)	229 (48)	0.956	0.328
Yes	15 (63)	9 (37)		
**Heard about e-cigarettes**				
No	88 (49)	90 (51)	1.231	0.267
Yes	178 (55)	148 (45)		
**Exposed to SHS regularly**				
No	124 (50)	123 (50)	0.843	0.358
Yes	115 (55)	96 (45)		
**Received cigarettes as gift or gave cigarettes as gifts to others**				
No	225 (53)	198 (47)	0.181	0.671
Yes	41 (51)	40 (49)		
**Hospital have any policy to advise smokers to quit**				
No	106 (48)	113 (52)	2.976	0.085
Yes	160 (56)	125 (44)		
**Received formal training in smoking cessation**				
No	199 (50)	200 (50)	6.716	0.010
Yes	62 (65)	34 (35)		
**Have read China smoking cessation guidelines**				
No	172 (53)	150 (47)	11.538	0.003
Yes	51 (66)	26 (34)		
Never heard about it	43 (41)	62 (59)		
**Have read international (i.e. US, UK) smoking cessation guidelines**				
No	193 (54)	166 (46)	2.711	0.258
Yes	22 (61)	14 (39)		
Never heard about it	51 (47)	58 (53)		
**Other international Guidelines are useful**				
No	3 (75)	1 (25)	3.972	0.137
Yes	20 (65)	11 (35)		
Not sure	1 (20)	4 (80)		
**Believe about the professional responsibility to discuss smoking cessation**				
Pediatricians	115 (50)	114 (50)	6.821	0.078
Nurses	82 (51)	78 (49)		
The parent’s primary care doctor	209 (55)	170 (45)		
Others:	64 (43)	86 (57)		
**Level of confidence discussing smoking cessation or SHS exposure reduction with patients’ parents**				
Not at all confident	25 (38)	41 (62)	6.764	0.009
Very/Somewhat confident	241 (55)	197 (45)		
**Believe pediatricians can help patients’ parents to stop smoking**				
Agree/strongly agree	188 (67)	93 (33)	50.849	0.000
Disagree/strongly disagree	78 (35)	145 (65)		
**I am not familiar with the guidelines for stop smoking**				
Agree/strongly agree	166 (54)	143 (46)	0.286	0.593
Disagree/strongly disagree	100 (51)	95 (49)		
**Smoking cessation counseling for my patients’ parents is not an efficient use of my time**				
Agree/strongly agree	130 (51)	126 (49)	0.832	0.362
Disagree/strongly disagree	136 (55)	112 (45)		
**I am unaware of the best strategies for helping my patients’ parents to stop smoking**				
Agree/strongly agree	169 (54)	144 (46)	0.490	0.484
Disagree/strongly disagree	97 (50)	94 (50)		
**Advise patients who smoke to avoid smoking around children**				
Agree/strongly agree	258 (55)	211 (45)	13.510	0.000
Disagree/strongly disagree	8 (23)	27 (77)		
**Health professionals should routinely ask about their patients smoking habits**				
Agree/strongly agree	237 (59)	166 (41)	29.351	0.000
Disagree/strongly disagree	29 (29)	72 (71)		
**Heath professionals should routinely advise their smoking patients to quit smoking**				
Agree/strongly agree	241 (61)	157 (39)	45.900	0.000
Disagree/strongly disagree	25 (24)	81 (76)		
**My current knowledge is sufficient for helping patients to stop smoking**				
Agree/strongly agree	64 (74)	23 (26)	18.228	0.000
Disagree/strongly disagree	202 (48)	215 (52)		
**I can assess a smoker’s different stages of readiness to quit**				
Agree/strongly agree	55 (67)	27 (33)	8.137	0.004
Disagree/strongly disagree	210 (50)	211 (50)		

Multinomial logistic regression analysis showed that “being confident in discussing smoking cessation or SHS exposure reduction with patients’ parents”, “believing that pediatricians can help parents stop smoking”, “believing that health professionals should routinely ask about their patients smoking habits”, “believing that health professionals should routinely advise their smoking patients to quit smoking”, “possessing adequate knowledge in quitting smoking”, and “being able to assess smokers different stages of readiness to quit” were the factors associated with the belief that pharmacological products are effective for smoking cessation (See Table 5).

**Table 5:** **Multinomial logistic regression analysis on factors associated with the belief that pharmacological products are effective for smoking cessation**

**Variables**	**Agree/strongly agree**	**Disagree/strongly disagree**	**Odds ratio**	***P*** **value**
	**n (%)**	**n (%)**	**(95% confidence interval)**	
**Level of confidence discussing smoking cessation or SHS exposure reduction with patients’ parents**				
Not at all confident	25 (38)	41 (62)	2.018	0.006
Very/Somewhat confident	241 (55)	197 (45)	(1.492–4.655)	
**Believe pediatricians can help patients’ parents to stop smoking**				
Agree/strongly agree	188 (67)	93 (33)	3.520	0.001
Disagree/strongly disagree	78 (35)	145 (65)	(1.731–7.924)	
**Health professionals should routinely ask about their patients smoking habits**				
Agree/strongly agree	237 (59)	166 (41)	3.685	0.000
Disagree/strongly disagree	29 (29)	72 (71)	(1.429–8.306)	
**Heath professionals should routinely advise their smoking patients to quit smoking**				
Agree/strongly agree	241 (61)	157 (39)	5.833	0.000
Disagree/strongly disagree	25 (24)	81 (76)	(2.072–11.448)	
**My current knowledge is sufficient for helping patients to stop smoking**				
Agree/strongly agree	64 (74)	23 (26)	4.156	0.000
Disagree/strongly disagree	202 (48)	215 (52)	(1.629–9.588)	
**I can assess a smoker’s different stages of readiness to quit**				
Agree/strongly agree	55 (67)	27 (33)	3.577	0.001
Disagree/strongly disagree	210 (50)	211 (50)	(1.605-8.184)	

## Discussion

To the best of our knowledge, this is the first study of pediatrician beliefs regarding the effectiveness of counseling and pharmacological products for smoking cessation in a developing country and in China. In the current study, 79% pediatricians did not receive any formal training on tobacco control or smoking cessation, although 84% of the pediatricians were very or somewhat confident about discussing smoking cessation or SHS exposure reduction with children’s parents. Studies elsewhere reported low rates of implementation of effective SHS exposure reduction interventions on parents who smoke among the pediatricians [16-18]. In an earlier study among Chinese parents [10], few parents (8/33, 24%) had positive experiences about the way they have been asked about SHS exposure to the children or about parental smoking status, and rarely were told by their child’s pediatricians to quit smoking.

Our findings show that about half of the pediatricians did not believe the fact that smoking cessation counseling and medication are effective in promoting smoking cessation. Evidence suggests that counseling [19,20] and cessation medications [21-25] are effective for smoking cessation. Factors that were associated with pediatricians’ beliefs included individual characteristics (i.e. female gender, perceived knowledge), clinical practice (i.e. receiving cessation training, advising to quit) and health system (i.e. hospital policy to ask about smoking) related factors. However, many other factors could influence these beliefs among the pediatricians. An earlier survey in China showed that, through creating smoke-free hospital activities, the rate of often asking patients’ smoking status increased from 55.0% to 68.9%, the rate of often advising patients to quit smoking from 67.8% to 77.3% [26]. The same study also reported higher confidence to provide smoking cessation service among physicians who received training on tobacco use prevention and cessation [26].

The low perceived effectiveness of counseling and medications for smoking cessation among Chinese pediatricians are not supported by the current evidence which suggests that these are effective intervention strategies [27-29]. The scarcity of smoking cessation services within the Chinese hospitals and the unavailability of cessation medications contributed significantly to possessing such beliefs among the pediatricians. It may be the fact that pediatricians were reluctant to know more about the effectiveness of these intervention modalities (i.e. counseling and medication) as they will not utilize these in their clinical practice. If these modalities for smoking cessation are to be fully implemented within the healthcare system, physicians need to be trained and aware of their effectiveness. At the same time, the Chinese healthcare system needs to realize the need for smoking cessation services with provision for medications. In this study, receiving formal training in smoking cessation or reading China smoking cessation guidelines were associated with the belief that cessation counseling and pharmacological products were effective.

## Limitations

Several factors may limit the generalizability of the findings. First, the sample may not be representative of the whole pediatric population in China. Second, although it is expected that the characteristics of pediatricians working in all the similar grade level hospitals would be similar, there may be regional variations. However, responses to key items did not differ as a function of hospital type or physician type so this is unlikely to have affected the results. Third, one might expect that attitudes would change over time as more information about the smoking cessation intervention was available in China since this study was conducted. A follow-up survey may examine the changes of beliefs over time.

## Conclusion

The findings suggest that a substantial proportion of Chinese pediatricians believed that cessation counseling and medications are not effective, which is not supported by current evidence in the field. Several factors including individual, practice level and health system level characteristics were associated with their belief regarding the effectiveness of cessation counseling and medications. Perceived effectiveness of cessation counseling and medication may affect pediatricians’ clinical practice and recommendations for tobacco use reduction and cessation. Therefore, training efforts are needed to influence pediatricians’ beliefs about the available evidence-based interventions (i.e., counseling and medications). At the same time, health system-level change to incorporate cessation service within the healthcare delivery system will increase pediatricians’ participation in the training, enhance their understanding about the evidence-based intervention available for smoking cessation and generate positive beliefs towards cessation counseling and medication.
